# Pomalidomide combinations are a safe and effective option after daratumumab failure

**DOI:** 10.1007/s00432-023-04637-x

**Published:** 2023-02-13

**Authors:** Annamaria Brioli, Laura Gengenbach, Katia Mancuso, Mascha Binder, Thomas Ernst, Florian H. Heidel, Thomas Stauch, Elena Zamagni, Inken Hilgendorf, Andreas Hochhaus, Monika Engelhardt, Marie von Lilienfeld-Toal

**Affiliations:** 1 Klinik für Innere Medizin C, Universitätsmedizin Greifswald, Greifswald, Germany; 2 Klinik für Innere Medizin II, Universitätsklinikum Jena, Jena, Germany; 3 Hämatologie und Onkologie, Faculty of Freiburg, Universität Freiburg, Freiburg, Germany; 4 Istituto di Ematologia “Seràgnoli”, IRCCS Azienda Ospedaliero-Universitaria di Bologna, Bologna, Italy; 5 Dipartimento di Medicina Specialistica, Diagnostica e Sperimentale, Università di Bologna, Bologna, Italy; 6 Klinik für Innere Medizin IV, Onkologie und Hämatologie, Martin-Luther-Universität Halle-Wittenberg, Halle (Saale), Germany

**Keywords:** Pomalidomide, Daratumumab, RRMM, Triple refractory, Extramedullary disease

## Abstract

**Purpose:**

Outcomes of multiple myeloma (MM) patients who are refractory to daratumumab are dismal and no standard of treatment exists for this patients’ population. Here, we investigate the role of pomalidomide combinations in daratumumab-refractory MM patients.

**Methods:**

We performed a retrospective analysis of myeloma patients treated at four referral centers (three in Germany and one in Italy). Review chart identified 30 patients with relapsed and refractory myeloma, who progressed during treatment with daratumumab and were treated with pomalidomide-based combinations in the subsequent lines of therapy.

**Results:**

Responses improved from 37% with daratumumab to 53% with pomalidomide. Of seven patients with extramedullary MM (EMM), four achieved a clinical stabilization with pomalidomide, including one patient with a long-lasting complete response. Median progression-free survival and overall survival were 6 and 12 months, respectively. Pomalidomide combinations were well tolerated, no patient discontinued treatment due to adverse events.

**Conclusion:**

These data show that pomalidomide-based combinations can be an effective and safe salvage regimen for daratumumab-refractory patients, including those with EMM.

**Supplementary Information:**

The online version contains supplementary material available at 10.1007/s00432-023-04637-x.

## Introduction

In the past decades, considerable advances have been made in the treatment of multiple myeloma (MM). The anti-CD38 monoclonal antibody daratumumab has shown remarkable activity in relapsed as well as in newly diagnosed MM patients and is now one of the preferred regimens both in the first line and at the time of relapse (Lonial et al. 2016; Palumbo et al. 2016; Dimopoulos et al. 2016, 2020; Facon et al. 2019; Moreau et al. 2019). Due to its recent approval and wide application, however, little is known about the best salvage treatment for patients who progress on daratumumab. Recent data indicate that outcome after relapse to daratumumab treatment is dismal (Gandhi et al. 2019), with a median overall survival (OS) shorter than 1 year. Survival is even worse for those patients refractory to an immunomodulator (IMiD), a proteasome inhibitor (PI), and a CD38 monoclonal antibody (triple refractory patients) (Mateos et al. 2022) and for those patients already exposed to two IMiDs, two PIs, and a CD38 monoclonal antibody (penta-exposed patients) (Gandhi et al. 2019). Pomalidomide is a third-generation IMiD approved for treatment of relapsed MM patients that have already been treated with lenalidomide and a PI. Pomalidomide alone and especially in combination has shown efficacy in relapsed/refractory multiple myeloma (RRMM) patients (San Miguel et al. 2013; Larocca et al. 2013; Dimopoulos et al. 2018; Richardson et al. 2019; Van Oekelen et al. 2020). The fact that pomalidomide is well tolerated in elderly and frail patients (Larocca et al. 2013), is important as patients with advanced disease frequently present in poor clinical condition. Here, we investigated the use of pomalidomide combinations in a cohort of patients progressing during daratumumab treatment to evaluate the role of pomalidomide combinations in this challenging patient population.

## Methods

Clinical records of patients treated at four University Hospitals, three in Germany (Jena, Halle and Freiburg), and one in Italy (Bologna), from 2016 to 2019, were analyzed retrospectively. All patients were followed until death or last follow-up, whichever occurred first. Patients lost to follow-up were censored at the date of the last follow-up. Progression-free survival (PFS) and OS were evaluated from the start of pomalidomide-based treatments using the Kaplan–Meier method. Data were analyzed using IBM SPSS, version 24. The study was approved by the institutional review boards.

## Results

Thirty pomalidomide-naïve patients treated with pomalidomide-based combinations after being refractory to daratumumab were identified. In 24 patients, pomalidomide was the first treatment administered after progression on daratumumab, while the remaining patients received one (five patients) to two (one patient) lines of therapy between daratumumab and pomalidomide. Patients’ characteristics are summarized in Table 1. Patients were heavily pretreated, with a median time between diagnosis and pomalidomide treatment of 5.5 years (range 1–17), and a median number of 4 previous lines of treatment (range 2–12). All patients were refractory to daratumumab. Twenty-three (77%) and sixteen out of thirty patients (53%) were refractory to IMiDs and PIs, respectively. Thirteen (43%) and five (16%) were triple refractory and penta exposed, respectively. Autologous stem cell transplantation had been performed in 23/30 (77%) patients, and 5/30 patients had also received an allogeneic stem cell transplantation. Seven patients had an extramedullary disease (EMM) at the time of pomalidomide treatment. Median number of cycles of daratumumab administered before receiving pomalidomide-based treatment was 4 (range 1–18). Responses to daratumumab were challenging in this intensively pretreated population: 27% of patients achieved a partial response (PR), 10% a very good PR (VGPR) and 17% had a stable disease (SD). No patient achieved a complete response (CR) and 43% were primary refractory.

**Table 1:** Patients’ characteristics at the time of starting pomalidomide

Parameter	*N* = 30
Patient-related data	
Age [median] (range) (years)	65 (47–84)
Male/Female (%)	16/14 (53%/47%)
MM-related data	
MM subtype [IgG/IgA/IgM/BJ/others], *n* (%)	13(43%)/7 (23%)/1(3%)/8(27%)/1(3%)
ISS Stage @diagnosis [I/II/III/NA], *n* (%)	7(23%)/10(33%)/8(27%)/5(17%)
EMM/PCL, *n* (%)	7 (23%)/1(3%)
High-risk cytogenetic^a^, *n* (%)	8 (27%)
Hemoglobin [median] (g/dl)	10.2 (6.8–15.9)
LDH [median] (U/l) (normal range < 253)	188 (132–1091)
Therapy-related data	
Previous lines of therapy [median] (range)	4 (2–12)
Previous daratumumab, *n* (%)	30 (100%)
Previous PIs, *n* (%)	29 (97%)
Previous IMiDs, *n* (%)	28 (93%)
Previous ASCT, *n* (%)	23 (77%)
Previous alloSCT, *n* (%)	5 (17%)
Refractory to daratumumab, *n* (%)	30 (100%)
Refractory to PI (%), *n* (%)	16 (53%)
Refractory to IMiD, *n* (%)	23 (77%)
Triple refractory (%), *n* (%)	13 (43%)
Penta exposed (%), *n* (%)	5 (16%)

*LDH* lactate dehydrogenase, *MM* multiple myeloma, *ISS* international staging system, *BJ* Bence Jones, *EMM* extramedullary myeloma, *PCL* plasma cell leukemia, *IMiDs* immunomodulatory drugs, *PIs* proteasome inhibitors, *ASCT* autologous stem cell transplantation, *alloSCT* allogeneic stem cell transplantation

^a^Includes *t*(4;14), *t*(14;16), del(17p), amp(1q)

Most patients received pomalidomide as part of a triplet combination. The drugs most frequently combined with pomalidomide were dexamethasone (29 cases) and cyclophosphamide (16 cases) (Garderet et al. 2018). Other combinations included elotuzumab (five cases) or PIs (two cases bortezomib, two cases carfilzomib, one case ixazomib). Three patients were treated only with the doublet pomalidomide/dexamethasone and one patient received pomalidomide monotherapy. No patient received daratumumab in combination with pomalidomide, as the combination of daratumumab, pomalidomide, and dexamethasone was not approved at the time. The median daily dose of pomalidomide was 4 mg (range 1–4) and the median monthly dose of dexamethasone was 80 mg (range 0–480 mg). For patients receiving cyclophosphamide, the median monthly dose of cyclophosphamide was 900 mg (range 150–1800 mg). The overall response rate [≥ PR] (ORR) was 53%, with 2/30 and 3/30 patients achieving a CR and a VGPR, respectively. The disease control rate (DCR  ≥ SD) was 83% (25/30 patients) (Supplementary Table 1). In those patients receiving pomalidomide combinations as first regimen after daratumumab failure, the ORR was 54% (13/24). Of the seven patients presenting with EMM, four out of seven achieved disease control (≥ SD), including three patients with a PR and one patient with a CR that lasted for more than 2 years. DCR for EMM patients increased from 28% (two out of seven patients) with daratumumab therapy to 86% (six out of seven patients) with pomalidomide combinations (Supplementary Table 2).

Responses to pomalidomide combinations were achieved promptly, with 60% of the patients responding within the first two cycles.

**Fig. 1:**
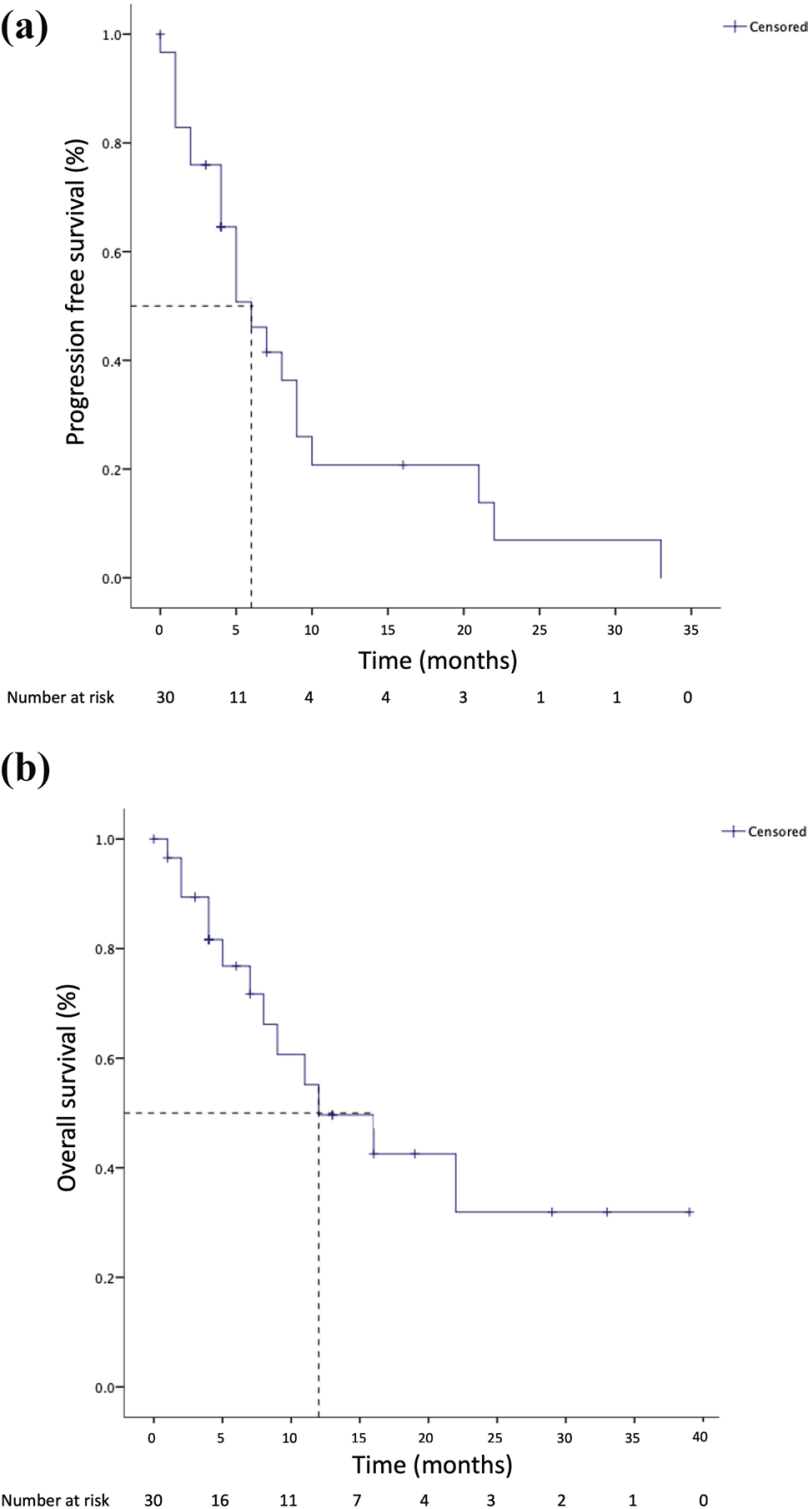
**a** Progression-free survival (PFS) landmarked at the start of pomalidomide-based combination. Median PFS 6 months [95% CI 3.4–8.6]; **b** overall survival (OS) landmarked at the start of pomalidomide-based combination. Median OS 12 months [95% CI 3.2–20.7]

With a median follow-up of 13 months (95% CI 3.3–22.7), the median PFS was 6 months (95% CI 3.4–8.5) and the median OS was 12 months (95% CI 3.3–20.7) (Fig. 1A and 1B).

Pomalidomide combinations were well tolerated. Neutropenia ≥ grade 3 was reported in five patients, of which only one developed a neutropenic fever. Two patients received prophylactic G-CSF. Two patients had a thrombocytopenia ≥ grade 3 and three patients had a grade 3 anemia. The most frequent non-hematologic adverse events (AEs) of grade ≥ 3 were infections (three pneumonia and two sepsis). Dose reductions due to AEs occurred in five patients, of which four dose-reduced pomalidomide.

## Discussion

The overall prognosis for patients with MM relapsing after salvage therapy with daratumumab appears disheartening (Gandhi et al. 2019). New treatment options are now available for these patients, including BCMA-immunoconjugates (Lonial et al. 2019), selinexor (Chari et al. 2019), CAR-T cells (Munshi et al. 2021; Berdeja et al. 2021), and bispecific antibodies (Moreau et al. 2022; Chari et al. 2022). Nevertheless, not all these treatments are widely available across countries or can be applied to all patients. Additionally, extensively treated RRMM often has an aggressive biology requiring combination therapy. Pomalidomide, a third-generation IMiD, is approved together with dexamethasone and can be applied in a variety of combinations for the treatment of RRMM. We here investigated the effect of pomalidomide-based regimens in patients refractory to daratumumab after a median of 4 lines of therapy. Overall, pomalidomide combinations were well tolerated and showed a significant response rate of 53% in this difficult-to-treat population. In comparison, Gandhi and colleagues reported an ORR of 31% to the first regimen given after daratumumab failure, identifying carfilzomib-based regimens and daratumumab plus IMiDs as treatments significantly associated with a longer PFS (Gandhi et al. 2019). More recently, the LocoMMotion study, a prospective, non-interventional, multinational study to assess the effectiveness of real-life standard of care treatments in triple-class exposed MM patients showed a median PFS of 4.6 months with a median OS of 12.4 months after diagnosis of the most recent relapse. Among the 248 patients enrolled, the ORR was 30%, with 12% of VGPR and only 1 patient achieving a CR (Mateos et al. 2022). Despite the limitation of a much smaller patients’ population and of the retrospective nature of our study, our results compare favorably with these data. Importantly, the LocoMMotion study showed that no standard treatment exists for triple-exposed RRMM patients, with 92 different combinations being reported in the trial, once more highlighting the unmet medical need of MM patients beyond the third line of therapy.

Among agents with a novel mechanism of action, selinexor (Chari et al. 2019) and belantamab-mafodotin (Lonial et al. 2019) have been reported to yield ORR between 26 and 30% in RRMM after failure of daratumumab. Due to the significantly different number of patients and the different trial design, no definitive conclusion can be drawn on what should be the preferred treatment regimen for patients refractory to daratumumab. Importantly, a recent phase 3 study failed to show a superiority of belantamab-mafodotin compared to pomalidomide in RRMM patients (GSK GmbH DREAMM-3 Announcement). CAR-T cells are highly effective, but their applicability is restricted to a limited number of patients. Similarly, bispecific antibodies require hospitalization for at least the first four doses. More than 90% of our study population had been exposed to IMiD or PI, with 43% of patients being triple refractory. Although the small number of patients prevented us from performing subgroup analyses, pomalidomide combinations were effective in this difficult-to-treat population, with a rate of disease control (at least SD) of 83%. Importantly, pomalidomide combinations were effective even in patients with extramedullary myeloma (EMM), with five out of seven patients with EMM achieving at least a clinical stabilization including one patient with a CR that lasted for more than 2 years (Supplementary Table 1).

At the time of treatment of our patients (between 2016 and 2019), the combination of pomalidomide, daratumumab, and dexamethasone was not approved in Europe and no patient in our cohort received treatment with pomalidomide combined with an anti-CD38 monoclonal antibody. Data from the Emory group on a limited number of patients showed that retreatment with daratumumab in combination with pomalidomide and dexamethasone can still be effective in patients that are daratumumab refractory (Nooka et al. 2019), suggesting that combining pomalidomide with an anti-CD38 antibody can add another treatment option for these patients.

In line with other reports (Larocca et al. 2013; Dimopoulos et al. 2018; Nooka et al. 2019), pomalidomide combinations were well tolerated, with AEs of grade ≥ 3 reported only in 17% of the patients. Hematologic adverse events were manageable, with only two patients receiving prophylactic G-CSF to maintain a neutrophil count above 1000/mm^3^. No patient had to discontinue treatment due to AEs.

Median PFS and OS compared well with those reported by MAMMOTH (Gandhi et al. 2019) and by the LocoMMotion (Mateos et al. 2022) studies, with 6 months and 12 months, respectively. Due to the limited number of patients included in this retrospective analysis, no further evaluation of the impact of high-risk cytogenetic profile or of the impact of EMM could be performed.

Although with the limitation of a small number of patients and the retrospective nature of our analysis from four large MM institutions, these data suggest that pomalidomide combinations are a helpful option for patients after several lines of therapy and refractory to daratumumab treatment. This concept is also illustrated by the various pomalidomide combinations recently approved, as well as the various combinations of pomalidomide with novel agents and immunotherapeutic approaches still under investigation in clinical trials. Due to the widespread use of daratumumab in all lines of therapy, the number of patients in this clinical situation is likely to increase. Here, treatment with a pomalidomide-containing combination is a well-tolerated option with beneficial efficacy. In the future, combinations of pomalidomide and drugs with a novel mechanism of action, such as bispecific antibodies, BCMA-immunoconjugates or selinexor may further improve patient outcome. Final data of ongoing studies with these combinations (NCT04484623, NCT02343042) are eagerly awaited and might change the treatment paradigm of RRMM patients.

In summary, pomalidomide-based treatment is a safe and effective option for patients refractory to daratumumab, supporting the use of these regimens as salvage treatment even in late lines of therapy.

## Supplementary Information

Below is the link to the electronic supplementary material.

Supplementary file1 (PDF 47 KB)

## Data Availability

The data supporting the findings of this paper are available from the corresponding author, AB, upon reasonable request.
