# Supplementary material for: Pomalidomide combinations are a safe and effective option after daratumumab failure
Source: J Cancer Res Clin Oncol. 2023 Feb 13;149(9):6569–74. doi: 10.1007/s00432-023-04637-x (PMC10356885; doi:10.1007/s00432-023-04637-x)
Supplement: Supplementary file 1 — Supplementary file1 (PDF 47 KB) [file 432_2023_4637_MOESM1_ESM.pdf]

SUPPLEMENTARY TABLES

Supplementary Table 1. Responses to daratumumab and pomalidomide combinations

**Responses**	**Responses to prior daratumumab combinations N=30 (%)**	**Current responses to pomalidomide combinations N=30 (%)**
CR	0	2 (7%)
VGPR	3 (10%)	3 (10%)
PR	8 (27%)	11 (37%)
MR	0	1 (3%)
SD	5 (17%)	8 (27%)
PD	13 (43%)	5 (17%)
NA	1 (3%)	0
ORR	11 (37%)	16 (53%)
CBR	11 (37%)	17 (57%)
DCR	16 (53%)	25 (83%)

CR: complete response; VGPR: very good partial response; PR: partial response; MR: minimal response; SD: stable disease; PD: progressive disease

ORR: overall response rate (≥PR); CBR: clinical benefit rate (≥MR); DCR: disease control rate (≥SD)

Supplementary Table 2. Responses to daratumumab and pomalidomide combinations in patients with extramedullary myeloma

Responses	Responses to prior daratumumab combinations N=7 (%)	Current responses to pomalidomide combinations N=7 (%)
CR	0	1 (14%)
VGPR	1 (14%)	0
PR	1 (14%)	3 (43%)
MR	0	1 (14%)
SD	0	1 (14%)
PD	5 (71%)	1 (14%)
ORR	2/7 (28%)	4/7 (57%)
CBR	2/7 (28%)	5/7 (71%)
DCR	2/7 (28%)	6/7 (86%)

CR: complete response; VGPR: very good partial response; PR: partial response; MR: minimal response; SD: stable disease; PD: progressive disease

ORR: overall response rate (≥PR); CBR: clinical benefit rate (≥MR); DCR: disease control rate (≥SD)
